# Value Assignment of Vitamin D and 25-Hydroxyvitamin D in Food-Matrix Standard Reference Materials (SRMs) Using Isotope Dilution Liquid Chromatography–Tandem Mass Spectrometry (ID LC–MS/MS)

**DOI:** 10.1021/acs.jafc.6c01298

**Published:** 2026-04-04

**Authors:** Carolyn Q. Burdette, James H. Yen, Adam J. Kuszak, Stephen A. Wise

**Affiliations:** † Chemical Sciences Division, National Institute of Standards and Technology (NIST), Charleston, South Carolina 29412, United States; ‡ Statistical Engineering Division, National Institute of Standards and Technology (NIST), Gaithersburg, Maryland 20899, United States; § Office of Dietary Supplements (ODS), National Institutes of Health (NIH), Bethesda, Maryland 20817, United States; ∥ IFC Contractor in Support of the Office of Dietary Supplements (ODS), National Institutes of Health (NIH), Bethesda, Maryland 20817, United States

**Keywords:** vitamin D, vitamin D metabolites, 25-hydroxyvitamin D_3_, vitamin D_2_, vitamin D_3_, cholecalciferol, ergocalciferol

## Abstract

Discrepancies exist between reported vitamin D dietary intake and measured status. Hydroxylated metabolites such as 25-hydroxyvitamin D [25­(OH)­D] may be more bioavailable; however, information on 25­(OH)­D content in foods is limited. To address this gap and support analytical method validation, NIST value assigned vitamin D_2_, vitamin D_3_, and 25-hydroxyvitamin D_3_ [25­(OH)­D_3_] in five food-matrix Standard Reference Materials® (SRMs). Using isotope dilution LC–MS/MS, values were established for SRMs 1546a (Meat Homogenate), 1549a (Whole Milk Powder), 1577c (Bovine Liver), 1845a (Whole Egg Powder), and 3235 (Soy Milk). Mass fractions ranged from 0.498 μg/kg to 49.5 μg/kg for vitamin D_3_ and 0.53 μg/kg to 12.1 μg/kg for 25­(OH)­D_3_. These SRMs enable validation of analytical methods and measurement comparability assessment across laboratories. By providing accurate reference values for both parent vitamins and metabolites, these materials support a better understanding of the relationship between dietary intake and overall nutritional status.

## Graphical abstract

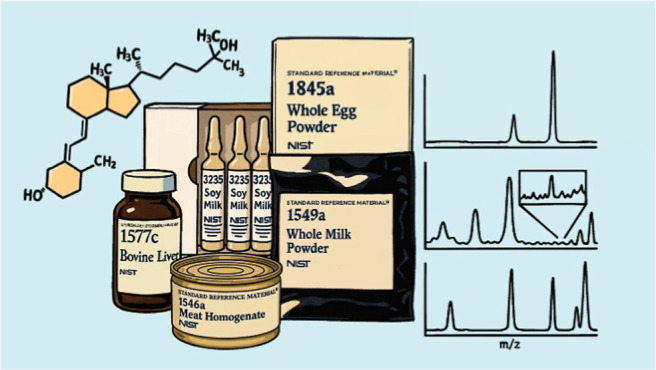

## Introduction

Vitamin D is a fat-soluble vitamin well understood for its importance in calcium absorption and bone metabolism, as well as other important roles in the body, including modulation of cell growth, regulation of immune function, and the reduction of inflammation.^1,2^ The two forms of vitamin D, vitamin D_2_ and vitamin D_3_, differ only in their side-chain structures. Vitamin D_3_ (cholecalciferol) is produced in the epidermis of the skin upon exposure to ultraviolet radiation (sunlight) and can also be obtained through dietary intake, whereas vitamin D_2_ (ergocalciferol) is obtained almost exclusively through supplementation. Both vitamin D_2_ and vitamin D_3_ undergo bioactivation, starting with hydroxylation in the liver to 25-hydroxyvitamin D_2_ [25­(OH)­D_2_] and 25-hydroxyvitamin D_3_ [25­(OH)­D_3_] and both have epimers that are present in human serum at about 5% of the concentration of the nonepimeric form.^3,4^ Clinically, vitamin D status is assessed by measuring the serum total 25­(OH)­D, which is the sum of 25­(OH)­D_2_ and 25­(OH)­D_3_ not including the 3-epimers. Determination of an individual’s vitamin D status is an important topic with significant current discussions and debate on the accuracy and comparability of test methods.^5–8^ Concerns about the accuracy of 25­(OH)­D measurements in clinical samples to assess vitamin D status have been addressed by improved methodologies,^9,10^ performance assessment schemes,^11,12^ and the use of calibration and validation tools such as certified reference materials (CRMs).^13,14^

Vitamin D activity derived from food is based primarily on the content of vitamin D_3_ with additional contributions attributed to vitamin D_2_ and/or 25­(OH)­D_3_.^15,16^ Only a limited number of foods contain endogenous vitamin D, primarily as vitamin D_3_, including fish liver oils, flesh tissue of fatty fish, and eggs among the best sources.^17,18^ Mushrooms are the only food source with significant endogenous vitamin D_2_ content.^19^ Fortified foods provide the most abundant source of consumed vitamin D in the U.S., where most milk and breakfast cereals and some brands of orange juice, yogurt, and margarine contain added vitamin D, typically as vitamin D_3_. Plant-based milk alternatives (e.g., soy, oat, or almond) are often fortified with vitamin D at an equivalent level as in fortified cow’s milk.

Discrepancies have been reported between vitamin D intake and the measured serum total 25­(OH)­D levels with serum levels generally higher than estimated based on dietary intake information.^15,16,18^ Differences in sun exposure, genetic polymorphisms in vitamin D metabolizing enzymes,^20,21^ underreporting of dietary intake, and potential differing bioavailability and nutritive value of vitamin D_3_ versus vitamin D_2_ ^22,23^ may all contribute to these observed discrepancies. However, the reliability of vitamin D dietary intake estimations remains a concern, specifically, the accurate measurement and inclusion of the 25­(OH)­D content in food sources. Recent reports have highlighted the challenges and knowledge gaps in assessing total vitamin D activity in food and the contribution of 25­(OH)­D to dietary status.^15,16,18^

Estimation of vitamin D dietary intake relies on international food composition databases^24,25^ to establish the total vitamin D content in dietary sources. Although these databases generally contain information on the vitamin D_3_ content in foods, significantly fewer database entries exist for 25­(OH)­D_3_. Currently, the U.S. Department of Agriculture’s (USDA) FoodData Central^26^ contains only eight unique food records that include values for 25­(OH)­D_3_ (egg, beef, and butter),^26^ while the Danish Frida food composition database^27^ currently reports the most extensive compilation of 25­(OH)­D content in foods with over 100 entries based on laboratory analyses. Food composition databases have traditionally applied a 5-fold activity factor to 25­(OH)­D_3_ in foods relative to vitamin D_3_,^28–30^ suggesting that 25­(OH)­D_3_ is more effective in increasing and maintaining 25­(OH)­D serum levels. However, there is a lack of international consensus. Jakobsen et al.^15,16^ have advocated that vitamin D vitamers should be regarded as equal in activity, and the vitamin D should be the sum of all vitamin D vitamers. Conversion factors in global databases have varied significantly, ranging from 1 to 5 for 25­(OH)­D and 1 to 2.5 for vitamin D_2_ and vitamin D_3_.^26,27^ The Danish Frida food composition database transitioned from using a factor of 5 to using 2.5, following European Food Safety Association (EFSA) recommendations.^16,27^ USDA Food Data Central does not include 25­(OH)­D_3_ in calculations of total vitamin D activity since its biological activity “has not been definitively determined”.^26^ These discrepancies underscore the critical need for accurate, harmonized measurement and reporting of all vitamin D content in foods to reconcile potential differences in bioavailability between isoforms and metabolites and provide consistent nutritional data.^15,16,31^

Analytical methods for the simultaneous determination of vitamin D_2_, vitamin D_3_, and 25­(OH)­D_3_ in foods and dietary supplements have been reported.^32–36^ However, until the work described in this report, few matrix-matched CRMs were available with values assigned for vitamin D_3_, and no food-matrix CRMs were available with values assigned for 25­(OH)­D_3_ content to validate analytical techniques and support the accuracy of these measurements. As part of the National Institutes of Health, Office of Dietary Supplements (NIH ODS) Vitamin D Initiative,^37^ an isotope dilution (ID) liquid chromatography–tandem mass spectrometry (LC–MS/MS) method was implemented to measure vitamin D_2_ or vitamin D_3_ and 25­(OH)­D_3_ in existing food-matrix CRMs and assign certified values. The following standard reference materials (SRMs), which are CRMs issued by NIST, were analyzed: SRM 1546a Meat Homogenate,^38^ SRM 1549a Whole Milk Powder,^39^ SRM 1577c Bovine Liver,^40^ SRM 1845a Whole Egg Powder,^41^ and SRM 3235 Soy Milk.^42^ The results obtained using this ID LC–MS/MS method were combined with results from a USDA-coordinated interlaboratory study^43^ in which several of these SRMs were analyzed by five laboratories with experience in measuring vitamin D and vitamin D metabolites in foods and dietary supplements and with measurements from other collaborators. The availability of food-matrix SRMs with values assigned for vitamin D_2_, vitamin D_3_, and 25­(OH)­D_3_ provides essential tools for validation of new analytical methodologies and ultimately to ensure the accuracy of food composition data used to estimate dietary intake.

## Materials and Methods

### Chemicals

Vitamin D_3_ (cholecalciferol), vitamin D_2_ (ergocalciferol), and 25-hydroxyvitamin D_3_ (25­(OH)­D_3_, as monohydrate) reference standards were acquired from United States Pharmacopeia (USP, Rockville, MD, USA). 25-Hydroxyvitamin D_2_ (25­(OH)­D_2_), 3-*epi*-25­(OH)­D_3_, and 3-*epi*-25­(OH)­D_2_ were obtained from Sigma-Aldrich (St. Louis, MO, USA). The isotopically labeled compounds vitamin D_3_-^13^C_5_, 25­(OH)­D_3_-^13^C_5_, and 3-*epi*-25­(OH)­D_3_-*d*_3_ were obtained from IsoSciences (King of Prussia, PA, USA). 4-Phenyl-1,2,4-triazoline-3,5-dione (PTAD), lipase (from *Candida rugosa*, Type VII), potassium phosphate (KH_2_PO_4_), butylated hydroxytoluene (BHT), dipotassium oxalate monohydrate, and magnesium sulfate (MgSO_4_) were obtained from Sigma-Aldrich (St. Louis, MO, USA). Potassium carbonate (K_2_CO_3_) was obtained from Fluka Chemika (Honeywell, Morris Plains, NJ). Phosphate buffer with a mass density of 2.72% was prepared with KH_2_PO_4_ and deionized water and adjusted to pH 7.9–8.0 using 50% KOH (aqueous). A 35% (mass density) dipotassium oxalate solution was prepared in deionized water. HPLC-grade acetonitrile, ethanol, ethyl acetate, hexane, 2-propanol, methanol, *tert*-butyl methyl ether (tBME), petroleum ether (PE), and water were used for solution preparation, sample preparation, and LC mobile-phase preparation.

### Standard Reference Materials

Food materials analyzed were existing SRMs produced and distributed by NIST (Gaithersburg, MD, USA) including SRM 1546a Meat Homogenate, SRM 1549a Whole Milk Powder, SRM 1577c Bovine Liver, SRM 1845a Whole Egg Powder, and SRM 3235 Soy Milk. SRM 1549a and SRM 3235 are fortified with vitamin D_3_ and vitamin D_2_, respectively, and the remaining SRMs contain only endogenous vitamin D_3_ and 25­(OH)­D_3_. SRM 1849a Infant/Adult Nutritional Formula (milk-based hybrid infant/adult nutritional powders), which is fortified with vitamin D_3_, was used as a control material for the determination of vitamin D_3_. SRM 3280 Multivitamin/Multielement Tablets (since replaced by SRM 3289 and SRM 3294) was used as a control material for vitamin D_2_ for the measurements in SRM 3235. No food matrix-matched control materials were available for 25­(OH)­D_3_ at the time of this study; therefore, SRM 1577c was used as a surrogate control material, based on a target value established in the USDA interlaboratory study.^43^

### Optimization of Digestion and Extraction

For sample preparation to release the fat-bound analytes from the food matrix, two different approaches were used: (1) a lipase-initiated enzymatic hydrolysis^44^ for SRM 1546a, SRM 1549a, and SRM 1577c and (2) a modified Röse–Gottlieb extraction with ethyl ether and petroleum ether in the presence of ammonia solution^45^ for SRM 1845a and SRM 3235. The lipase approach is an alternative to analyte release under milder conditions, thereby minimizing the potential for analyte degradation. In both approaches, the released fat-soluble vitamins are then isolated from the matrix through precipitation and liquid–liquid extraction (LLE).

The amount of lipase required and the number of LLE cycles required for complete extraction were investigated. Lipase optimization was initially performed using SRM 1546a. Eight 2 g subsamples were treated with varying lipase amounts ranging from approximately 0.1 to 1.0 g, followed by four hexane LLE extraction cycles. The mass fractions of vitamin D_3_ and 25­(OH)­D_3_ were determined using ID LC–MS/MS, and the amounts measured did not significantly increase above an addition of 0.7 g of lipase, as illustrated in Figure S1 (Supporting Information). Similar findings were observed in an additional study using a SRM 1549a. Since the fat contents of SRM 1549a and SRM 1577c are comparable to or lower than that of SRM 1546a, the addition of 0.7 to 1.0 g of lipase per 2 g sample was used for all subsequent value assignment measurements.

The number of LLE cycles was evaluated for each SRM matrix to ensure an exhaustive recovery of vitamin D_3_ and 25­(OH)­D_3_. To isolate extraction efficiency from enzymatic digestion effects, an excess of lipase (1.5 g per 2 g sample) was used during these optimization studies. For hexane-based extractions (SRMs 1546a, 1577c, and 1549a), each cycle consisted of adding 20 to 30 mL of hexane, followed by 15 min of sonication, 30 min of rotary mixing, and 10 min of centrifugation. Two-to-seven cycles were tested, with the first volume extracted overnight, and it was observed that analyte mass fractions did not increase significantly after the fourth cycle for SRM 1546a and SRM 1577c and after the third cycle for SRM 1549a (Figure S2, Supporting Information). The extraction of vitamin D_3_ and 25­(OH)­D_3_ from SRM 1845a and vitamin D_2_ from SRM 3235 focused on the number of tBME:PE extraction volumes. Comparison of four and six volumes demonstrated that there was no increase in levels of vitamin D_3_ or 25­(OH)­D_3_ after four extractions (Figure S2, Supporting Information); therefore, four volumes were used for the final analysis of these materials.

### Sample Preparation

To minimize the potential for analyte degradation, all sample preparation steps were completed in subdued lighting, with samples shielded from light when possible. Prior to sample preparation, each sample was thoroughly homogenized. Ten packages of each food-matrix SRM, which were selected using a stratified random sampling scheme, and two packets of SRM 1849a and 1 bottle of SRM 1577c (as control materials) were prepared in duplicate on subsequent days for analysis.

For SRMs 1546a, 1549a, and 1577c, a 2 to 3 g subsample was accurately weighed into a vessel and appropriate volumes of internal standard solutions were gravimetrically added to match the target value of each analyte. The enzymatic digestion was initiated with addition of 20 mL of phosphate buffer (mass density of 2.7%) and ≈1.0 g lipase per 2 g of food material and then incubated at 40 °C for 2 h while stirring. Samples were cooled to room temperature, and a 10 mL aliquot of ethanol (containing ≈30 mg/g BHT) and 1.0 g of potassium carbonate were added. Analytes were extracted from the matrix by adding 20 to 30 mL of hexane (containing ≈30 mg/g BHT) and stirring overnight (15–18 h) at room temperature. The samples were then transferred to 50 mL polypropylene tubes and centrifuged (1450*g*_*n*_) for 10 min. The top organic layers were transferred to clean polypropylene tubes. An additional 20 to 30 mL aliquot of hexane was added to each sample, and the tubes were placed in an ultrasonic bath for 30 min (no heat), followed by rotary mixing for 30 min. The samples were again centrifuged for 10 min, and the organic layers were combined with the previous volumes. The process was repeated for a total of 3 to 4 extractions, depending on the matrix. MgSO_4_ was added to the pooled organic layers, which were then vortexed for 1 min, centrifuged (2000*g*_*n*_) for 10 min, and dried under N_2_ at 45 °C. The analytes were derivatized by adding 200 μL of PTAD solution (≈2.5 mg/g in acetonitrile) and 400 μL of acetonitrile. The samples were vortexed for 30 s and allowed to stand at room temperature for 30 min. The PTAD solution should be a bright-red/pink color when added. As the PTAD is consumed, either by derivatization or degradation, the pink color dissipates. After 30 min, an additional 200 μL of PTAD solution was added, and the samples were vortexed for 30 s and allowed to stand at room temperature for another 30 min. Samples were dried under N_2_ (at 45 °C) and reconstituted with a solution of 400 to 500 μL of methanol and ethyl acetate, with a volume fraction of 50% methanol. Particulate matter was removed by centrifugation at 161,000*g*_*n*_ for 10 min, and 400 μL of each sample was transferred to an amber autosampler vial with a 500 μL glass insert.

For SRM 1845a and SRM 3235, ≈2 g of sample material was weighed into a 50 mL polypropylene centrifuge tube, and appropriate volumes of internal standard solutions were gravimetrically added by mass to match the target value of each analyte. The sample and internal standards were suspended in ≈5 mL of HPLC-grade water and shaken until the sample had visibly dispersed. A 0.2 mL aliquot of a dipotassium oxalate aqueous solution (mass density of 35%) was added to the sample, followed by 10 mL of ethanol and 12 mL of a solution of tBME and PE, with a volume fraction of 58% PE. The sample was mixed with rotational agitation for 30 min and then centrifuged at 1450*g*_*n*_ for 5 min. Following centrifugation, the upper tBME:PE phase was transferred to a clean 50 mL polyethylene centrifuge tube, and the lower phase was extracted with another 12 mL aliquot of fresh tBME:PE. This process was repeated for a total of four extractions, and the tBME:PE fractions were combined. MgSO_4_ was added to the pooled organic layers, which were then vortexed for 1 min, centrifuged (2000*g*_*n*_) for 10 min, and dried under N_2_ at 45 °C. Derivatization with PTAD was performed as described above for the other SRMs.

The sample preparation steps described were not optimized for green chemistry and broad adoption but to value assign reference materials. These reference materials can be used to help validate other techniques, including those that focus on green analytical principles.

### ID LC–MS/MS Analysis

LC Instrumental Method 1: The LC separation was performed on an Ascentis Express F5 (pentafluoropropyl) column (Supelco (Bellefonte, PA), 5 cm × 4.6 mm, 2.7 μm particles) with an F5 guard column with the same stationary phase material and temperature controlled at 30 °C with a flow rate of 1.1 mL/min. Mobile-phase component A was water, and mobile-phase component B was methanol. The gradient elution began with a 1 min hold at 70% B and then 70% B to 95% B over 11 min, immediately followed by 5 min at 100% B and a 5 min equilibration at the initial conditions.

LC Instrumental Method 2: LC separation was achieved by a gradient method using a Pack Pro C_18_ RS (YMC, 5 cm × 4.6 mm, 3 μm particles) equipped with an F5 guard column, temperature controlled at 30 °C with a flow rate of 1.0 mL/min. Gradient elution began with 75% B for 10 min, 75% B to 95% B over 10 min, followed by a 5 min wash at 100% B and a 5 min equilibration at the initial conditions. Mobile-phase component A was water, and mobile-phase component B was methanol.

MS/MS Instrumental Method: Mass Spectrometric detection was performed using an AB Sciex API 5000 LC–MS/MS system (Sciex, Framingham, MA) equipped with an Agilent 1260 Series LC instrument (Agilent Technologies, Santa Clara, CA). Analyte ionization was achieved with atmospheric pressure chemical ionization (APCI) and the ions were detected with positive polarity in multiple reaction monitoring (MRM) mode. The parameters for each MRM transition (Table 1) were as follows: dwell time 200 ms, curtain gas 276 kPa (40 psi), collision gas 55 p*K*_a_ (8 psi), ion source gas1 517 p*K*_a_ (75 psi), needle current 5 μA, source temperature 375 °C, declustering potential 60 V, entrance potential 13 V, collision energy 15 V, and exit potential 20 V. Mass spectral data were acquired using Analyst software (v 1.6.2; Sciex). Blank injections were monitored for sample carry-over. Injection volumes were 5 μL for samples and calibrants and 10 μL for blanks. The autosampler tray temperature was set between 15 and 20 °C depending on the sample matrix.

**Table 1:** MRM Transitions Used for the APCI LC–MS/MS Method

analyte	precursor ion (*m*/*z*)	precursor ion (*m*/*z*)
25(OH)D_3_	558	298
3-*epi*-25(OH)D_3_	558	298
25(OH)D_3_-^13^C_5_	563	298
3-*epi*-25(OH)D_3_-*d*_3_	561	298
25(OH)D_2_	570	298
3-*epi*-25(OH)D_2_	570	298
vitamin D_3_	560	298
vitamin D_3_-^13^C_5_	565	298
vitamin D_2_	572	298

Analyte mass fractions of vitamin D and 25­(OH)­D were determined in each food material with three to six independently prepared calibration solutions. Stock solutions of each analyte and internal standard were prepared in ethanol and then gravimetrically mixed with a 1:1 mass ratio of unlabeled to labeled compound at representative levels of each of the samples. The mixtures were dried under N_2_ at ≈45 °C and derivatized by adding 200 μL of PTAD working solution, vortexing for 30 s, and standing at room temperature for 30 min to 1 h (depending on the time required for the food samples). The derivatized calibrants were then dried and reconstituted with 500 μL of methanol and ethyl acetate (80:20, v/v) or 500 μL of ethanol containing 30 ppm of BHT, depending on the corresponding sample preparation used. After centrifugation (161,000*g*_n_) for 10 min, calibrants were transferred to amber autosampler vials. Each calibration solution was injected in duplicate, and peak areas were used to calculate response factors for each analyte for each injection. % CVs for the response factors were <6% for 25­(OH)­D_3_, <4% for vitamin D_3_, and 16% for vitamin D_2_, which were used to calculate analyte concentrations in each sample. The limits of detection (LoDs) and limits of quantification (LoQs) were calculated by using a single-point approach for each matrix.

### Analysis of Control Materials

For the analysis of each food-matrix SRM batch, SRM 1849a was included as a control material for the determination of vitamin D_3_. SRM 1577c was included as a control material for the determination of 25­(OH)­D_3_ for SRM 1549a and SRM 1845a since an appropriate reference material was not available (not included for SRM 1546a). Since SRM 1577c was used as a control material before a certified value was assigned, the consensus mean value of 0.0151 mg/kg ± 0.00021 mg/kg (SD) (see Table 3) from the USDA interlaboratory study^43^ was used as a target value. For the determination of vitamin D_2_ in the soy milk (SRM 3235), SRM 3280 Multivitamin/Multielement Tablets was used as a control material.

## Results and Discussion

The goal was to implement a state-of-the-art ID LC–MS/MS method to simultaneously measure vitamin D_2_, vitamin D_3_, 25­(OH)­D_2_, 25­(OH)­D_3_, and 3-*epi*-25­(OH)­D_3_ with sufficient sensitivity and selectivity to determine endogenous levels in food matrices. The first analytical methods for vitamin D and 25­(OH)­D in foods were based on LC with UV detection after saponification, extraction, and chromatographic separation using vitamin D_2_ as an internal standard for vitamin D_3_ and 25­(OH)­D_3_.^34,35,46,47^ Recent methods developed for vitamin D and 25­(OH)­D in food matrices have been based on LC with MS or MS/MS detection,^36,48^ and some methods for simultaneously determining vitamin D and 25­(OH)­D are based on methods developed for 25­(OH)­D in serum and have been adapted for use with food matrices.^33,49^ For example, Burild et al.^32,33^ developed an ID LC–MS/MS method for simultaneous determination of vitamin D_3_, 25­(OH)­D_3_, and 24,25­(OH)_2_D_3_ in human serum using PTAD derivation, SPE cleanup, separation on a C_18_ stationary phase column, and deuterated analogues of vitamin D_3_ and 25­(OH)­D_3_ as internal standards. The quantification principle developed for serum was combined with the extraction procedure commonly used for food matrices, specifically saponification,^35^ and applied to porcine fat and liver samples.^34^

After analyte release, through either the lipase enzymatic hydrolysis or modified Röse–Gottlieb protocol and liquid–liquid extraction (LLE), a PTAD derivatization time of 30 min was used to ensure full derivatization of the analytes in the complex food extract matrices. Two different LC separations were evaluated using a PFP (Method 1) and a C_18_ (Method 2) stationary phase as illustrated in Figure 1.

**Figure 1:**
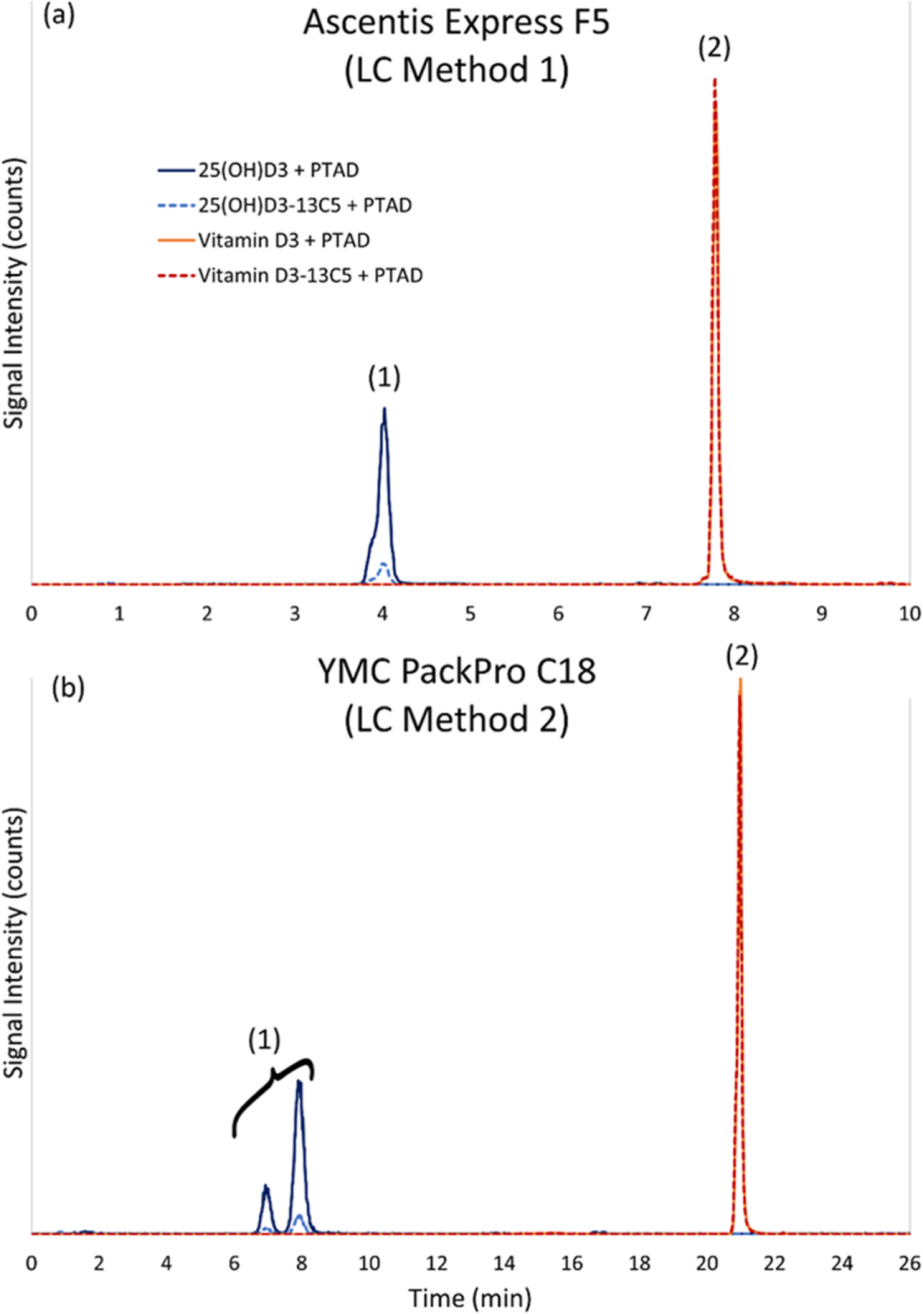
Representative chromatograms for the analysis of a calibrant solution containing vitamin D_3_ and 25­(OH)­D_3_ after derivatization with PTAD using (a) LC Method 1 on a pentafluorophenyl (PFP) column and (b) LC Method 2 on a C_18_ column. Peak 1 is 25­(OH)­D_3_ + PTAD (solid dark-blue trace, 558 *m*/*z* → 298 *m*/*z*) and 25­(OH)­D_3_-^13^C_5_ + PTAD (dashed light-blue trace, 563 *m*/*z* → 298 *m*/*z*). Note that in LC Method 2, (b) the 25­(OH)­D_3_ and the 25­(OH)­D_3_-^13^C_5_ + PTAD are each two chromatographic peaks. Peak 2 is vitamin D_3_ + PTAD (solid orange trace, 560 *m*/*z* → 298 *m*/*z*) and vitamin D_3_-^13^C_5_ + PTAD (dashed red trace, 565 *m*/*z* → 298 *m*/*z*).

Method 1 using the PFP stationary phase achieved separation of the 25­(OH)­D_3_-PTAD and vitamin D_3_-PTAD in less than 10 min (Figure 1a), whereas the C_18_ column achieved the same separation in 22 min (Figure 1b) and provides a better separation of the 3-*epi*-25­(OH)­D_3_-PTAD from the 25­(OH)­D_3_-PTAD (Figure 2).

**Figure 2:**
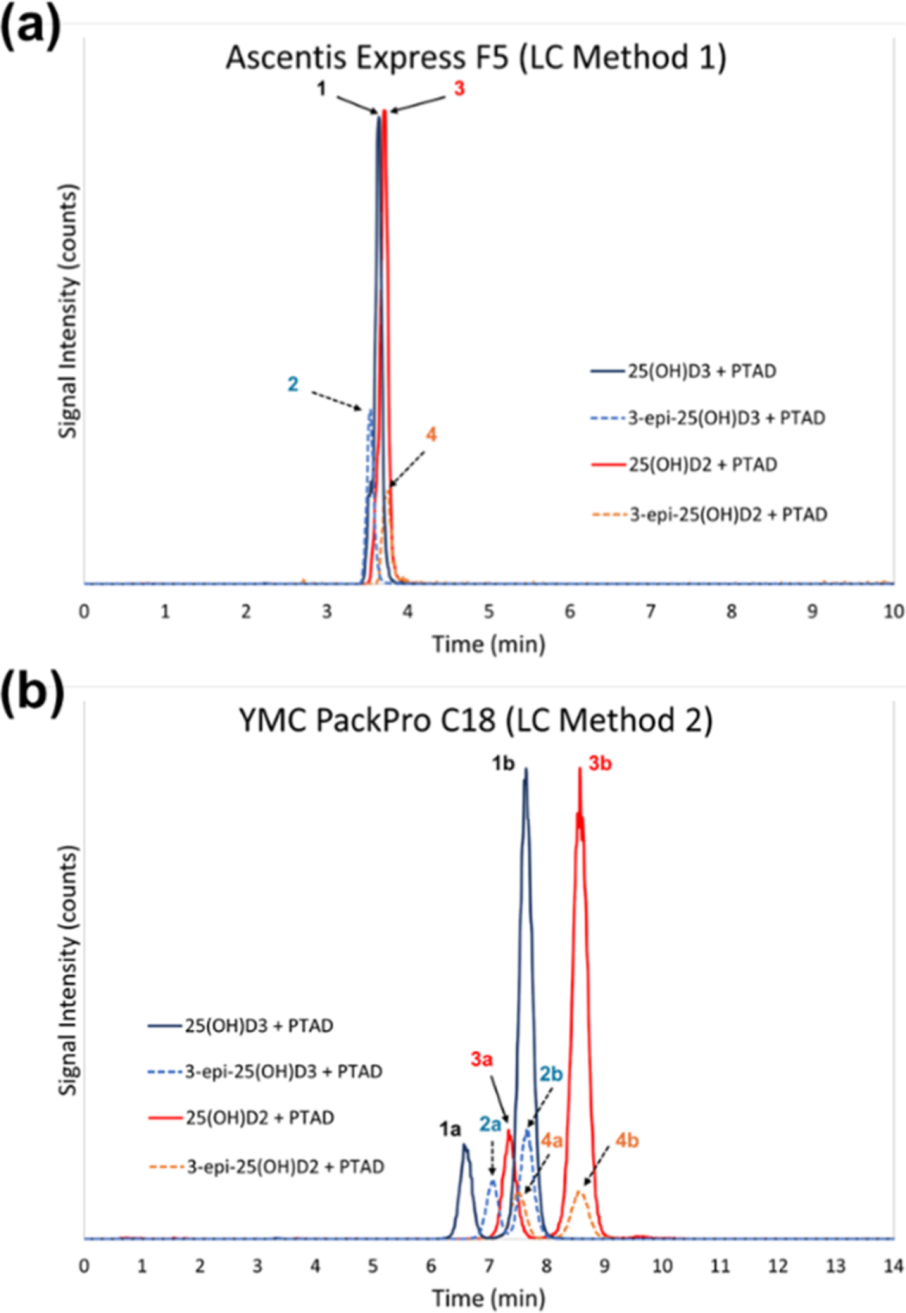
Representative chromatograms for the analysis of a calibrant solution containing 25­(OH)­D_2_, 25­(OH)­D_3_, 3-*epi*-25­(OH)­D_2_, and 3-*epi*-25­(OH)­D_3_ after derivatization with PTAD using (a) LC Method 1 on a pentafluorophenyl (PFP) column and (b) LC Method 2 on a C_18_ column. Peaks 1 and 2 in (a) and 1a and 1b and 2a and 2b in (b) are 25­(OH)­D_3_ + PTAD (solid dark-blue trace, 558 *m*/*z* → 298 *m*/*z*) and 3-*epi*-25­(OH)­D_3_ + PTAD (dashed light-blue trace, 563 *m*/*z* → 298 *m*/*z*), respectively. Peaks 3 and 4 in (a) and 3a and 3b and 4a and 4b in (b) are 25­(OH)­D_2_ + PTAD (solid dark-blue trace, 570 *m*/*z* → 298 *m*/*z*) and 3-*epi*-25­(OH)­D_2_ + PTAD (dashed light-blue trace, 570 *m*/*z* → 298 *m*/*z*), respectively. Note that in LC Method 2, (b) the 25­(OH)­D_3_-^13^C_5_ + PTAD is two chromatographic peaks.

As shown in Figures 1b and 2b, during the derivatization, 6­(*R*) and 6­(*S*) enantiomers of the 25­(OH)­D_3_-PTAD and 25­(OH)­D_3_-^13^C_5_-PTAD are formed in a 1:4 ratio as reported previously,^50–52^ and they are separated on the C_18_ column but not on the PFP column. The separation of the PTAD enantiomers for 25­(OH)­D_2_, 25­(OH)­D_3_, 3-*epi*-25­(OH)­D_2_, and 3-*epi*-25­(OH)­D_3_ is illustrated in Figure 2b for the C_18_ column. When the C_18_ column is used, chromatographic peaks for both enantiomers must be quantified. For the nonfortified food matrices investigated, only vitamin D_3_ and 25­(OH)­D_3_ were present in quantifiable amounts, and 3-*epi*-25­(OH)­D_3_ was not detected. Vitamin D_2_ and 25­(OH)­D_2_ were observed in SRM 1577c and 1546a; however, the measurement variability was significant, and the results were not used for value assignment. Therefore, Method 1, with its shorter analysis time and the lack of detectable 3-*epi*-25­(OH)­D_3_, was used for the analyses of these food-matrix SRMs to assign concentration values for vitamin D_3_ and 25­(OH)­D_3_.

Initial method development utilized deuterated internal standards for vitamin D_3_ and 25­(OH)­D_3_; however, hydrogen–deuterium exchange was observed following PTAD derivatization. This was observed by the detection of unlabeled MRM transitions in solutions containing exclusively labeled standards, as shown in Figure S3 (Supporting Information). The exchange was notably more significant for vitamin D_3_-*d*_3_ than that for 25­(OH)­D_3_-*d*_6_. In contrast, the analysis of ^13^C_5_-labeled compounds showed no isotopic loss or exchange. While the exact timing of the exchange, whether during chemical derivatization, reconstitution, LC solvent exposure, or within the mass spectrometer, remains unconfirmed, these findings suggested a transition to ^13^C_5_-labeled internal standards to ensure analytical robustness. It is understood that other methods have successfully used deuterium-labeled internal standards, but the potential of this deuterium/hydrogen exchange should be considered when developing new methods for vitamin D and metabolites.^53^

### Analysis of Food-Matrix SRMs for Determination of Vitamin D and 25­(OH)­D_3_

The ID LC–MS/MS method was used to determine both vitamin D_3_ and 25­(OH)­D_3_ in three nonfortified food matrices (meat homogenate, bovine liver, egg powder) and in a single fortified matrix (whole milk powder) and to determine vitamin D_2_ in a single fortified matrix (soy milk). Three of these SRMs (bovine liver, meat homogenate, and whole egg powder) were identified as high-priority food-matrix SRMs for vitamin D measurements based on the results of the USDA interlaboratory study.^43^ Representative chromatograms from the ID LC–MS/MS analysis of the four food-matrix SRMs and the control material (SRM 1849a) for the determination of vitamin D_3_ and 25­(OH)­D_3_ are shown in Figure 3, and the results of the analysis are summarized in Table 2. As observed in the USDA Interlaboratory Study,^43^ the bovine liver has the highest content of 25­(OH)­D_3_ and the lowest content of vitamin D_3_ of the four matrices investigated, while the whole egg material has high content for both vitamin D_3_ and 25­(OH)­D_3_. The mass fractions in the four matrices for vitamin D_3_ range over 2 orders of magnitude from 0.498 to 49.5 μg/kg and for 25­(OH)­D_3_ range a factor of 25 from 0.53 to 12.1 μg/kg. The application of the ID LC–MS/MS method for determining vitamin D_2_ is illustrated in Figure 4 for the analysis of SRM 3235 Soy Milk with SRM 3280 Multivitamin/Multielement Tablets as the control material.

**Figure 3:**
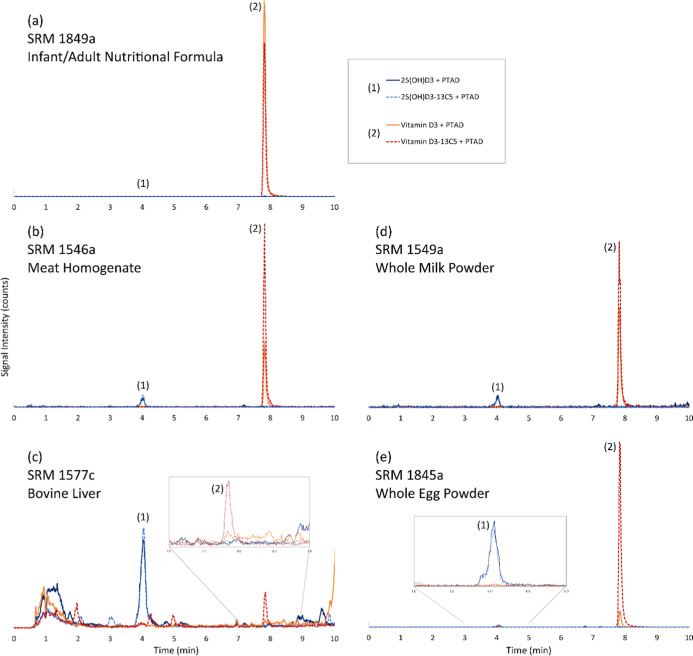
Representative chromatograms using LC method 1 for the analysis of (a) SRM 1849a Infant/Adult Nutritional Formula, (b) SRM 1546a Meat Homogenate, (c) SRM 1577c Bovine Liver, (d) SRM 1549a Whole Milk Powder, and (e) SRM 1845a Whole Egg Powder. Peak 1 is 25­(OH)­D_3_ + PTAD (solid dark-blue trace, 558 *m*/*z* → 298 *m*/*z*) and 25­(OH)­D_3_-^13^C_5_ + PTAD (dashed blue trace, 563 *m*/*z* → 298 *m*/*z*). Peak 2 is vitamin D_3_ + PTAD (solid orange trace, 560 *m*/*z* → 298 *m*/*z*) and vitamin D_3_-^13^C_5_ + PTAD (dashed red trace, 565 *m*/*z* → 298 *m*/*z*).

**Table 2:** Determination of Vitamin D_3_, Vitamin D_2_, and 25­(OH)­D_3_ in Five Food-Matrix SRMs Using the ID LC–MS/MS Method^d^

	mass fraction, μg/kg^a^ (as-received basis)									
samples		SRM 1577c bovine liver		SRM 1546a meat homogenate		SRM 1845a whole egg powder		SRM 1549a whole milk powder		SRM 3235 soy milk
box^b^	rep^c^	vitamin D_3_	25(OH)D_3_	vitamin D_3_	25(OH)D_3_	vitamin D_3_	25(OH)D_3_	vitamin D_3_	25(OH)D_3_	vitamin D_2_
1	1	0.399	13.01	2.24	0.75	49.16	11.07	1.536	0.596	13.9
1	2	0.307	11.99	3.75	1.54	50.97	10.62	1.616	0.537	13.7
2	1	0.711	12.90	2.73	1.01	47.03	11.35	1.676	0.636	12.5
2	2	0.994	14.07	2.35	0.73	59.67	11.95	2.095	0.511	11.6
3	1	0.604	12.90	3.41	0.74	46.76	11.60	1.802	0.539	11.4
3	2	0.992	12.52	2.05	0.80	53.17	9.85	2.149	0.579	11.5
4	1	0.613	11.64	8.89	0.55	47.67	11.05	1.908	0.558	10.7
4	2	0.300	10.87	2.09	0.81	51.75	11.26	1.943	0.459	11.1
5	1	0.939	12.57	1.75	0.90	46.71	11.09	2.086	0.469	11.8
5	2	1.906^c^	12.52	2.29	0.67	46.78	10.59	1.901	0.543	11.4
6	1	0.324	12.99	1.82	0.90	48.73	11.59	1.850	0.592	11.8
6	2	0.289	12.03	3.46	0.89	47.19	10.96	2.882^c^	0.473	12.4
7	1	0.777	18.98^c^	2.13	0.84	48.60	9.25	1.903	0.450	10.7
7	2	0.335	11.29	4.56	0.57	50.54	11.26	2.016	1.090^c^	10.5
8	1	0.255	10.88	1.81	0.65	48.57	10.88	1.807	0.673	11.2
8	2	0.262	10.08	2.53	0.92	53.07	9.23	1.598	0.603	11.3
9	1	0.299	12.48	1.86	1.12	46.95	11.36	0.794^c^	0.600	10.5
9	2	0.433	12.09	2.69	0.77	54.29	12.20	2.075	0.177	10.7
10	1	0.337	13.85	2.10	0.71	45.01	11.18	1.726	0.583	11.7
10	2	0.294	10.06	1.84	0.94	47.88	10.70	1.928	0.571	11.8
**mean**		**0.498**	**12.14**	**2.82**	**0.84**	**49.52**	**10.95**	**1.867**	**0.534**	**11.6**
**SD**		**0.263**	**1.12**	**1.61**	**0.22**	**3.48**	**0.77**	**0.183**	**0.106**	**0.9**
**% RSD**		**52.8%**	**9.2%**	**57.3%**	**25.9%**	**7.0%**	**7.1%**	**9.8%**	**19.8%**	**8.1%**

a Results are presented in units of μg/kg for presentation convenience rather than in mg/kg as the assigned values in Table 3.

b Box numbers in table are only a sequential designation of the 10 boxes sampled; actual box numbers are as follows: SRM 1577c (1, 4, 7, 10, 13, 16, 19, 22, 25, and 28); SRM 1546a (1, 38, 52, 111, 151, 240, 247, 297, 379, and 484); SRM 1845a (1B, 1T, 2B, 3B, 3T, 4B, 5B, 5T, 6B, and 6T where B = bottom and *T* = top of box); SRM 1549a (1, 3, 6, 10, 12, 16, 20, 24, 27, and 33); and SRM 3235 (1, 3, 5, 6, 7, 9, 10, 15, 17, and 19).

c Duplicate sample preparation and analysis.

d Replicate measurement was determined to be an outlier based on the Grubbs test and result not used in the value assignment of the SRM.

**Figure 4:**
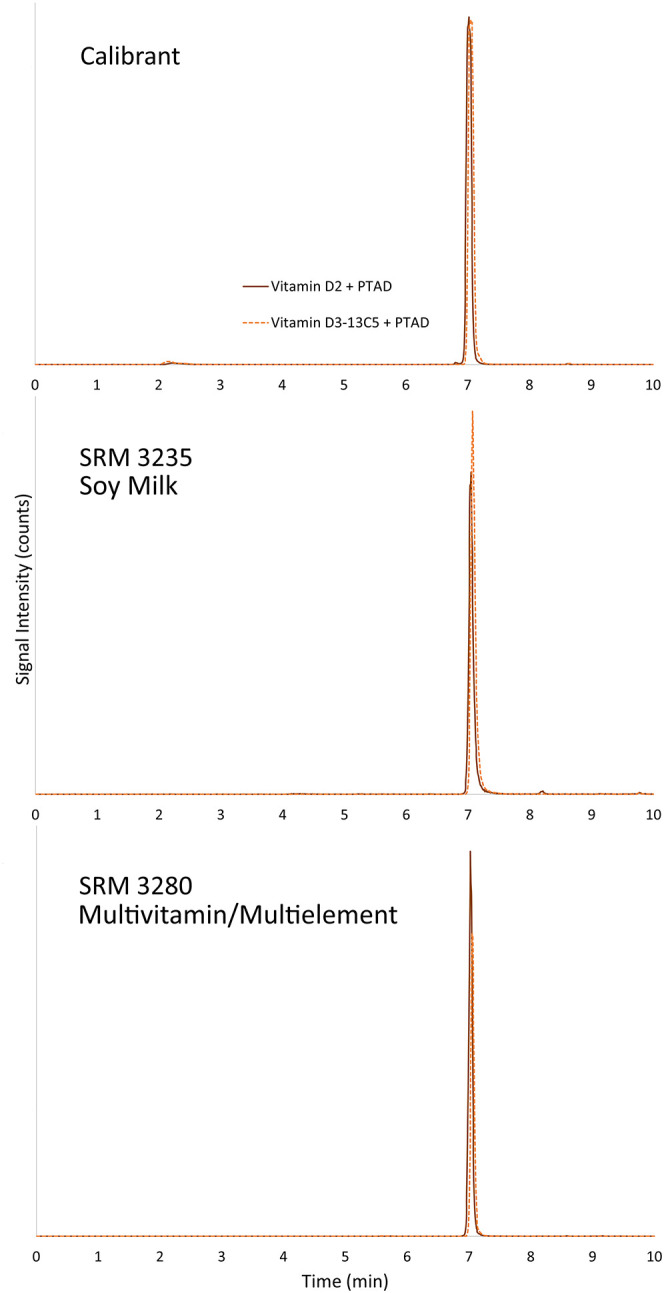
Representative chromatograms using LC method 1 for the analysis of (a) a calibrant solution containing vitamin D_2_-PTAD and vitamin D_3_-^13^C_5_ + PTAD, (b) SRM 3235 Soy Milk, and (c) SRM 3280 Multivitamin/Multielement Tablets (control material). Solid orange trace is vitamin D_2_ + PTAD (572 *m*/*z* → 298 *m*/*z*) and dashed orange trace is vitamin D_3_-^13^C_5_ + PTAD (565 *m*/*z* → 298 *m*/*z*).

The results in Table 2 represent measurements of 10 different samples in duplicate, which reflect both the homogeneity of the different food-matrix SRMs and demonstrate the precision of the ID LC–MS/MS method. For the two powdered matrices (egg and milk) and the liquid soy milk, the %RSDs for the vitamin D measurements were between 7.0% and 9.8%. For the bovine liver and meat homogenate matrices, the %RSDs were 53% and 57%, respectively, demonstrating the complexity and relative inhomogeneity of these matrices, as well as their challenging low mass fractions of vitamin D_3_ (particularly in the bovine liver) and 25­(OH)­D_3_. When the measurements were evaluated relative to the selection of samples across the complete inventory of SRM units related to packaging, sample preparation, or run order, no trends were observed, as illustrated for SRM 1845a and SRM 1549a in Figures S4 and S5 (Supporting Information).

For the determination of vitamin D_3_ in the food-matrix SRMs, SRM 1849a Infant/Adult Nutritional Formula was used as a control material, and the results for the four SRMs were all within (or with overlapping SD) the certified value as shown in Figure 5 and Table S1 (Supporting Information).

**Figure 5:**
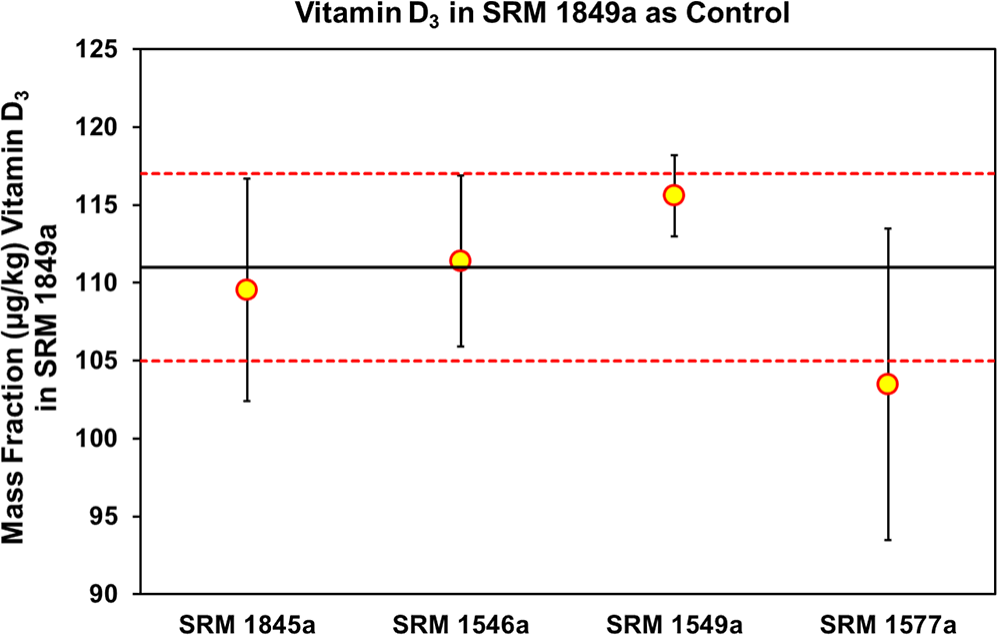
Results for the analysis of the SRM 1849a Infant/Adult Nutritional Formula as a control material during the analysis of food-matrix SRMs for determining vitamin D_3_. Certified value for vitamin D_3_ is a solid black line and the uncertainty of the certified value is represented by the dashed red line. Yellow circles represent the mean of the 4 to 8 measurements, and the error bars represent ± SD of the measurements.

Since no control material was available with a value assigned for 25­(OH)­D_3_ in a food matrix, SRM 1577c Bovine Liver was used as a control material using the consensus mean value obtained from the USDA Interlaboratory Study^43^ as the target control value, i.e., 0.0151 mg/kg ± 0.0021 mg/kg (SD). Results for the analyses of SRM 1577c as a control material for determination of 25­(OH)­D_3_ are shown in Figure S6 and Table S1 (Supporting Information). For the 25­(OH)­D_3_ control measurements, the measurements overlapped the SD of the target value for two of the three SRMs (black lines, Figure S6, Supporting Information). However, after assignment of a certified value for 25­(OH)­D_3_ (red lines, Figure S6, Supporting Information) using these measurements and the USDA consensus value, results are all within the uncertainty of the assigned value. For the analysis of the soy milk SRM, which contains vitamin D_2_ rather than vitamin D_3_, SRM 3280 Multivitamin/Multielement Tablets was used as the control material and the mean result was within the uncertainty of the certified value (Figure S7, Supporting Information).

The mean values of the NIST measurements, the mean from collaborative data, and the resulting certified and reference values assigned for vitamin D_2_, vitamin D_3_, and 25­(OH)­D_3_ are provided in Table 3.

**Table 3:** Mass Fraction Values for Vitamin D_3_ and 25­(OH)­D_3_ from a USDA Interlaboratory Study and NIST in This Study and the Resulting Values Assigned in the SRM Certificate of Analysis^i^

SRM	description	USDA interlab study (SD)^a^	this study (SD)^b^	assigned value^c^
Vitamin D_3_ (Cholecalciferol) Mass Fraction (mg/kg) As-Received Basis				
1546a	meat homogenate	0.00233 (0.00034)	0.00282 (0.00161)	0.00256 ± 0.00053 (21%)^d^
1549a	whole milk powder	ND	0.00187 (0.00018)	0.00188 ± 0.0035 (19%)^e^
1577c	bovine liver	0.00043 (0.00025)	0.000498 (0.000263)	0.000464 ± 0.000363 (78%)^f^
1845a	whole egg powder	0.0449 (0.0045)	0.0495 (0.0035)	**0.0488 ± 0.0047** **(10%)** ^g^
25(OH)D_3_ Mass Fraction (mg/kg) As-Received Basis				
1546a	meat homogenate	0.00093 (0.00019)	0.0084 (0.0022)	**0.00090 ± 0.00012** **(13%)** ^g^
1549a	whole milk powder	ND	0.000534 (0.00011)	0.00053 ± 0.00005 (9%)^e^
1577c	bovine liver	0.0151 (0.0021)	0.01214 (0.00112)	**0.0137 ± 0.0031** **(23%)** ^g^
1845a	whole egg powder	0.0125 (0.0020)	0.01095 (0.00077)	**0.0122 ± 0.0015** **(12%)** ^g^

		collaborating laboratories^h^	this study (SD)^b^	assigned value
Vitamin D_2_ (Ergocalciferol) Mass Fraction (mg/kg) As-Received Basis				
3235	soy milk	0.0127 (0.0006)	0.0116 (0.0009)	**0.0120 ± 0.0024** (20%)^g^

a USDA interlaboratory study consensus value from results from four to five laboratories using LC–MS/MS,^43^ which was combined with the NIST measurements to assign certified values. SD = standard deviation of the four to five results in parentheses.

b SD = standard deviation of the 10 samples × 2 replicate measurements in Table 2.

c Assigned values reported on the Certificate of Analysis for each SRM; numbers in bold are certified values, whereas numbers in normal face type are reference values; number in parentheses is the percent relative uncertainty.

d Each reference mass fraction value is the mean from the combination of mean of results from analyses by NIST and mean of the means of results provided by collaborating laboratories. Values are expressed as *x* ± U95% (*x*), where *x* is the reference value and U95% (*x*) is the expanded uncertainty of the value. The method-specific value of the analyte lies within the interval *x* ± U95% (*x*) with about a 95% confidence.

e The reference mass fraction value is the mean result of NIST analyses. Values are expressed as *x* ± U95% (*x*), where x is the reference value and U95% (*x*) is the expanded uncertainty of the value. The method-specific value of the analyte lies within the interval *x* ± U95% (*x*) with about a 95% confidence.

f No value for vitamin D_3_ in SRM 1577c was assigned and reported on the COA. The value in the table is for information only and is the mean of the collaborating laboratory value and the NIST measurements with a combined expanded uncertainty.

g The certified mass fraction value is the mean from the combination of the mean results from analyses by NIST ID LC–MS/MS and the median or mean of the means of results provided by collaborating laboratories. Values are expressed as *x* ± U95% (*x*), where *x* is the certified value and U95% (*x*) is the expanded uncertainty of the certified value. The true value of the analyte lies within the interval *x* ± U95% (*x*) with 95% confidence. To propagate this uncertainty, the certified value should be treated as a normally distributed random variable with mean *x* and standard deviation U95% (*x*)/2.^55–58^

h Collaborating laboratories in interlaboratory study reported methods included LC-absorbance, LC–MS, and LC–MS/MS. Results reported are from Grocery Manufacturers Association Food Industry Analytical Chemists (FIAC) Share Group results; outliers removed. *n* = 5, uncertainty is standard deviation.

i ND = not determined.

These certified and reference values were added to the Certificate of Analysis for these SRMs between 2016 and 2018 with a brief description of the analytical methods.^38–42^ Although no values were assigned for vitamin D_2_ or 25­(OH)­D_2_ in SRM 1546a and SRM 1577c due to significant variability in the measurements, these two materials could be useful for these measurements. In the USDA interlaboratory trial, a consensus mean value for 25­(OH)­D_2_ of 0.00064 mg/kg (SD = 0.00015 mg/kg, *n* = 5) (as-received basis) was reported for SRM 1577c.^43^ Cashman et al.^48^ recently reported measurable levels of both vitamin D_2_ and 25­(OH)­D_2_ in beef and lamb steaks and used SRM 1546 Meat Homogenate to validate measurements of vitamin D_3_ and 25­(OH)­D_3_. The importance of accurate measurements of all vitamin D and 25­(OH)­D species was emphasized by the statement that “one-third of the total vitamin D activity of Irish beef was attributable to its combined vitamin D_2_ and 25­(OH)­D_2_ content, estimates of which are largely or completely missed in food composition tables”.^48^ Since the assignment of values for vitamin D_3_ and 25­(OH)­D_3_ in food-matrix SRMs reported in this paper, Švarc et al.^54^ investigated extensively the quantification of vitamin D_3_ and 25­(OH)­D_3_ in food and the effect of eluent additives and the use of labeled internal standards in the LC–MS/MS methods using three food-matrix SRMs (SRM 1546a, SRM 1845a, and SRM 1549a) for method validation and comparison. In comparing three ID LC–MS/MS methods with both deuterated and ^13^C-labeled internal standards, they found the method using ^13^C-labeled to be the preferred method.^54^

With the addition of vitamin D_3_ and 25­(OH)­D_3_ values in these food matrix SRMs ranging from 0.498 to 49.5 μg/kg and 0.53 to 12.1 μg/kg, respectively, these SRMs have and will continue to assist researchers in assessing the accuracy of vitamin D measurements in food.

## Supplementary Material



## Notes

Additional tables and figures, including method optimization results, internal standard hydrogen–deuterium exchange, and control material analyses (PDF)
