# Supplementary material for: Value Assignment of Vitamin D and 25-Hydroxyvitamin D in Food-Matrix Standard Reference Materials (SRMs) Using Isotope Dilution Liquid Chromatography–Tandem Mass Spectrometry (ID LC–MS/MS)
Source: J Agric Food Chem. 2026 Apr 4;74(18):14607–18. doi: 10.1021/acs.jafc.6c01298 (PMC13178072; doi:10.1021/acs.jafc.6c01298)

Supporting Information

Value Assignment of Vitamin D and 25-Hydroxyvitamin D in Food-Matrix Standard Reference Materials (SRMs) using Isotope Dilution Liquid Chromatography - Tandem Mass Spectrometry (ID LC-MS/MS)

Carolyn Q. Burdette[1*], James H. Yen[2], Adam J. Kuszak[3], and Stephen A. Wise[4]

[1] Chemical Sciences Division, National Institute of Standards and Technology (NIST), Charleston, SC 29412, carolyn.burdette@nist.gov, *Corresponding author

[2] Statistical Engineering Division, National Institute of Standards and Technology (NIST), Gaithersburg, MD 20899, james.yen@nist.gov

[3] Office of Dietary Supplements (ODS), National Institutes of Health (NIH), Bethesda, MD 20817, adam.kuszak@nih.gov

[4] IFC contractor in support of the Office of Dietary Supplements (ODS), National Institutes of Health (NIH), Bethesda, MD 20817, stephen.wise@nih.gov

Table S1. Determination of Vitamin D_3, Vitamin D_2, and 25(OH)D_3 in SRM 1849a, SRM 1577c, and SRM 3280 as Control Materials

		SRM 1577c Bovine Liver		SRM 1546a Meat Homogenate		SRM 1845a Whole Egg Powder		SRM 1549a Whole Milk Powder		SRM 3235 Soy Milk
		**SRM 1849a**	**SRM 1577c**	**SRM 1849a**	**SRM 1577c**	**SRM 1849a**	**SRM 1577c**	**SRM 1849a**	**SRM 1577c**	**SRM 3280**
		**Mass Fraction, µg/kg[a]**								
**ID[b]**	**Prep[c]**	**Vitamin D_3**	**25(OH)D_3[d]**	**Vitamin D_3**	**25(OH)D_3[e]**	**Vitamin D_3**	**25(OH)D_3**	**Vitamin D_3**	**25(OH)D_3**	**Vitamin D_2**
A	1	90		110		118.9	14.11	112.8	12.44	8.7
A	2	114		114		106.9	11.07	114.2	11.71	9.75
A	1					97.0	14.10	119.5	12.19	
A	2					107.5	13.18	114.9	12.05	
A	1						15.36			
A	2						12.87			
A	1						17.03			
A	2						13.01			
B	1	104		103		107.6		116.8		6.91
B	2	106		118		113.6				7.62
B	1			112		118.1		112.4		
B	2					106.7		114.0		
**Mean**		**103.5**		**111.4**		**109.5**	**13.84**	**115.6**	**12.10**	**8.25**
**SD**		**1.0**		**5.5**		**7.15**	**1.79**	**2.6**	**0.30**	**1.25**
**% RSD**		**9.6**		**5.0**		**6.5**	**12.9**	**2.2**	**2.5**	**15.1**
**n**		**4**		**5**		**8**	**8**	**7**	**4**	**4**

[a] Results are presented in units of µg/kg for presentation convenience rather than in mg/kg as the assigned values in Table 3.

[b] Two packets of SRM 1849a were used (A and B) with multiple subsamples from each packet; one bottle of SRM 1577c (A) was used with multiple subsamples from the one bottle; two bottles of SRM 3280 (A and B) were used with duplicate subsamples from each bottle.

[c] Duplicate sample preparations from each subsample.

[d] Bovine liver was not run as a control during the analysis of the bovine liver for 25(OH)D_3; see results in Table 2.

[e] SRM 1577c was not analyzed during the analysis of SRM 1546a.

Figure S1. Summary of extraction study using SRM 1546a Meat Homogenate and varying amounts of lipase, each followed by four hexane LLE extraction cycles.

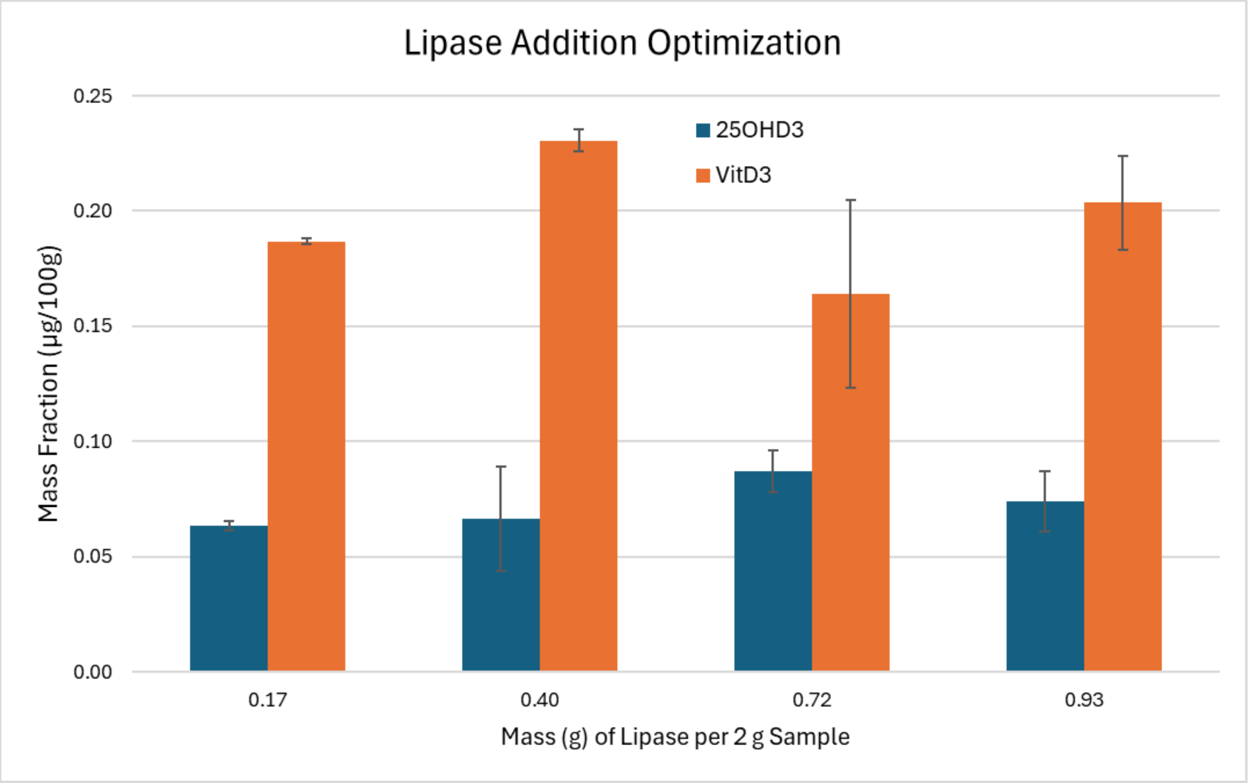

Figure S2. Summary of extraction study focused on number of LLE extraction cycles. For SRM 1577c, SRM 1546a, and SRM 1549a the extraction solvent was hexane. For SRM 1845a and SRM 3235 the extraction solvent was tBME:PE.

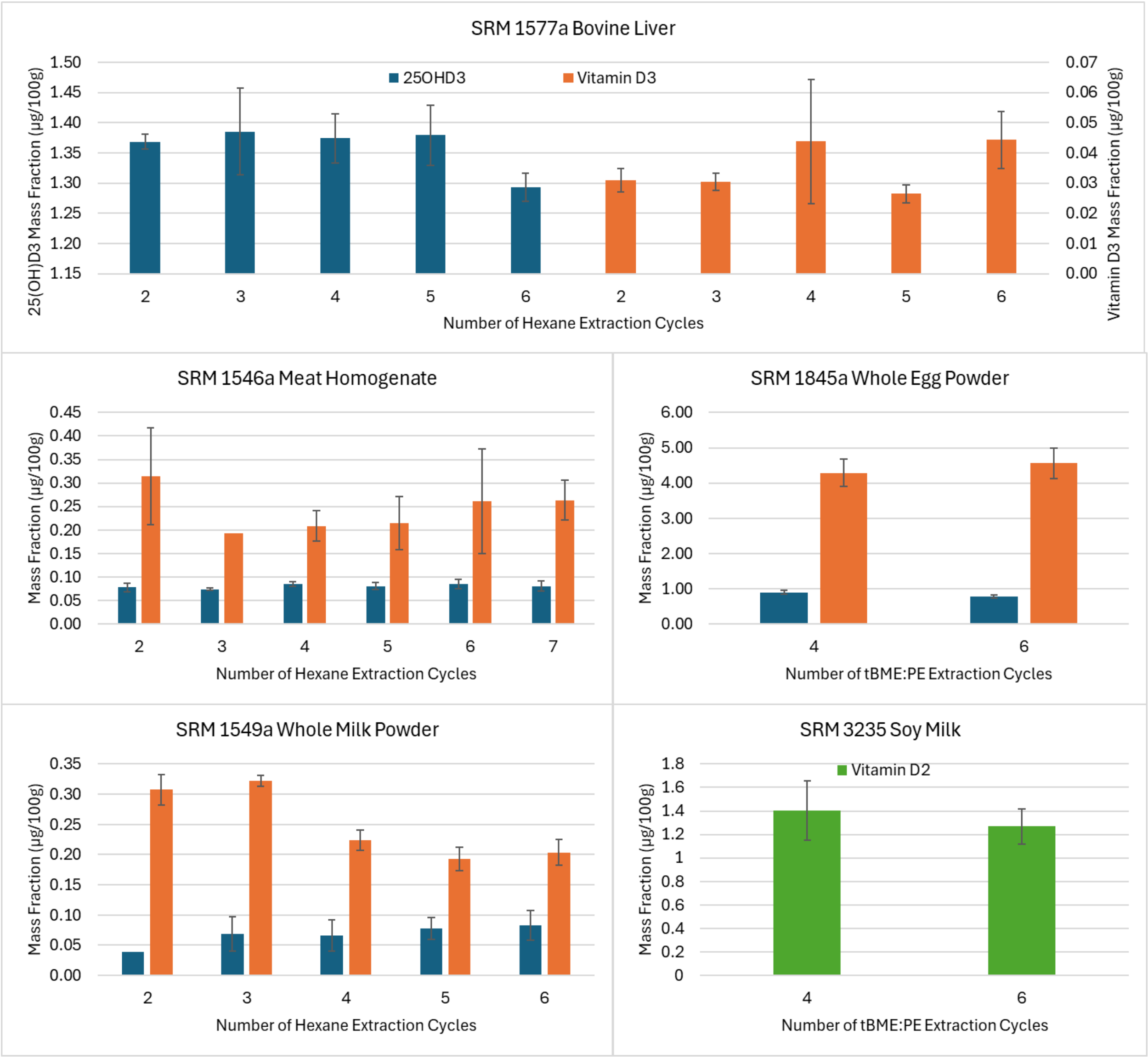

Figure S3. Left: Selected ion chromatograms for *m/z* 564 → 298 [25(OH)D_3-d_6 + PTAD] and *m/z* 558 → 298 [25(OH)D_3 + PTAD] of a solution of only 25(OH)D_3-d_6 derivatized with PTAD. Right: Selected ion chromatograms for *m/z* 563 → 298 [25(OH)D_3-$^{13}C_5$ + PTAD], and *m/z* 558 → 298 [25(OH)D_3 + PTAD] of solution of only 25(OH)D_3-$^{13}C_5$ derivatized with PTAD.

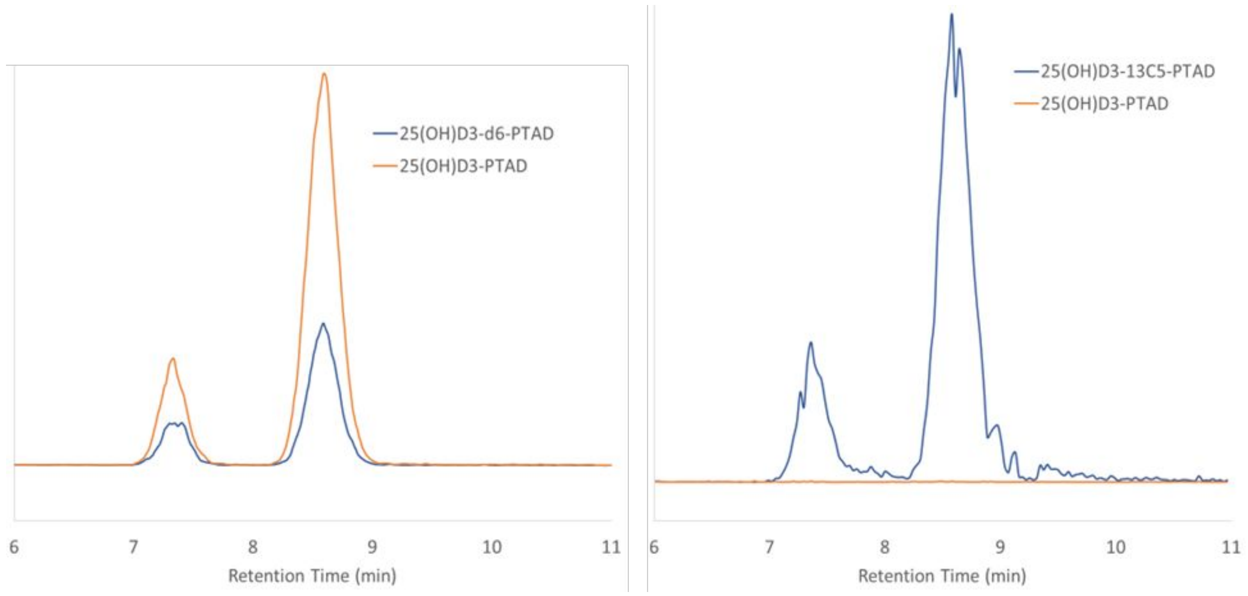

Figure S4. ID LC-MS/MS measurements of (A) vitamin D_3 (cholecalciferol) and (B) 25(OH)D_3 in SRM 1845a Egg Powder as a function of packaging order (box number). Dots colors represent different days for sample preparation (blue = day 1, red = day 2). Error bars are ± SD of combined measurements of results from method 1 and method 2.

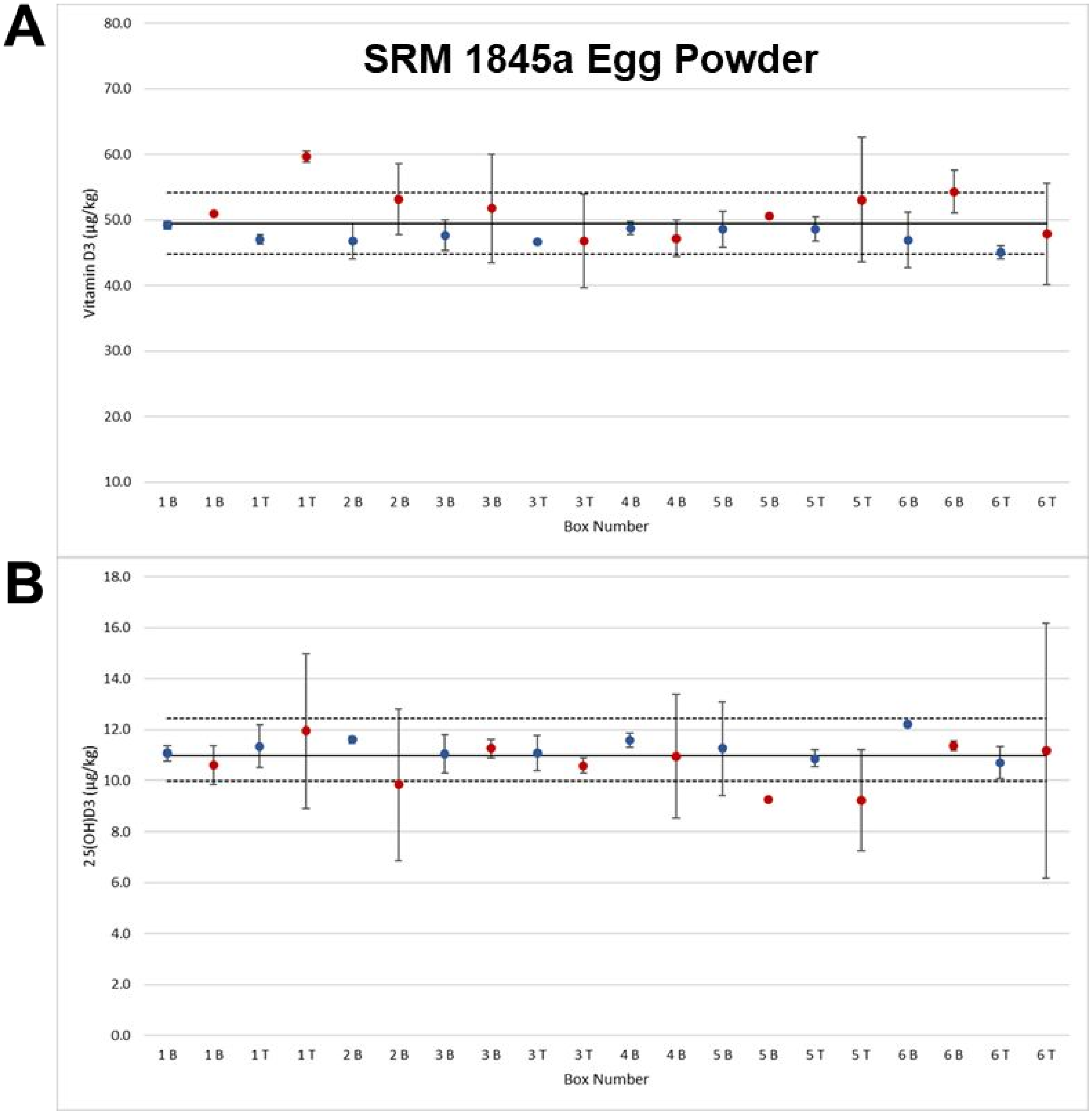

Figure S5. ID LC-MS/MS measurements of (A) vitamin D_3 (cholecalciferol) and (B) 25(OH)D_3 in SRM 1549a Whole Milk Powder as a function of packaging order (box number). Dot colors represent different days for sample preparation (blue = day 1, red = day 2). Error bars (as ± 1 SD) for vitamin D_3 are from combined measurements of results from method 1 and method 2. Only Method 2 was used for value assignment of 25(OH)D_3.

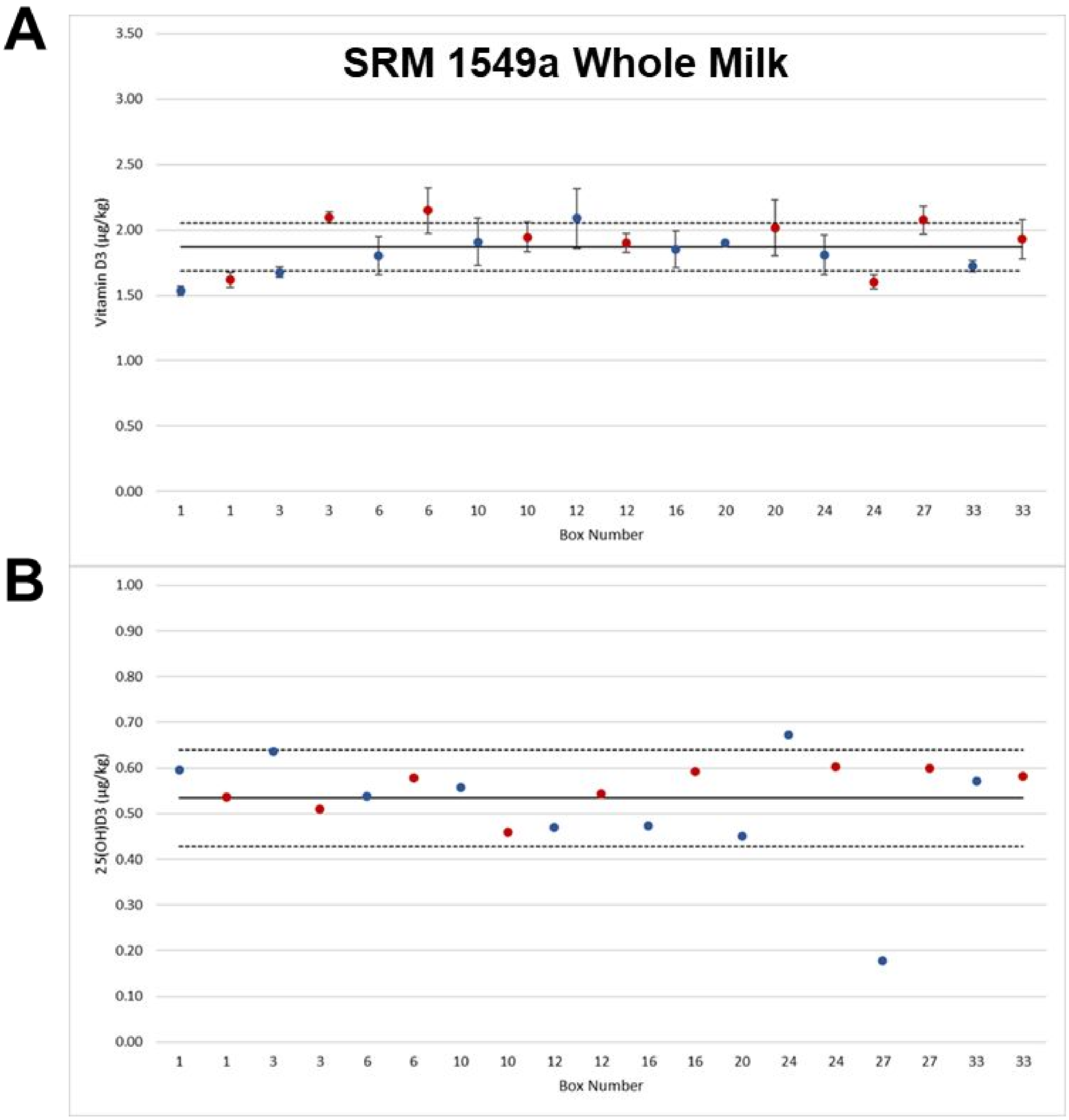

Figure S6. Results for the analysis of SRM 1577c Bovine Liver as control material during the analysis of food-matrix SRMs for the determination of 25(OH)D_3. Consensus value from USDA study for 25(OH)D_3 is solid black line and dashed black line represents ±SD of the consensus value. The solid red line and the dashed red lines represent the assigned certified value and associated uncertainty, respectively, for 25(OH)D_3 in SRM 1577c. Yellow circles represent the mean of the 4 and 8 measurements of SRM 1577c when analyzed concurrently with SRM 1549a and SRM 1845a, respectively, and the mean of 20 measurements for SRM 1577c; error bars represent ±SD of the measurements.

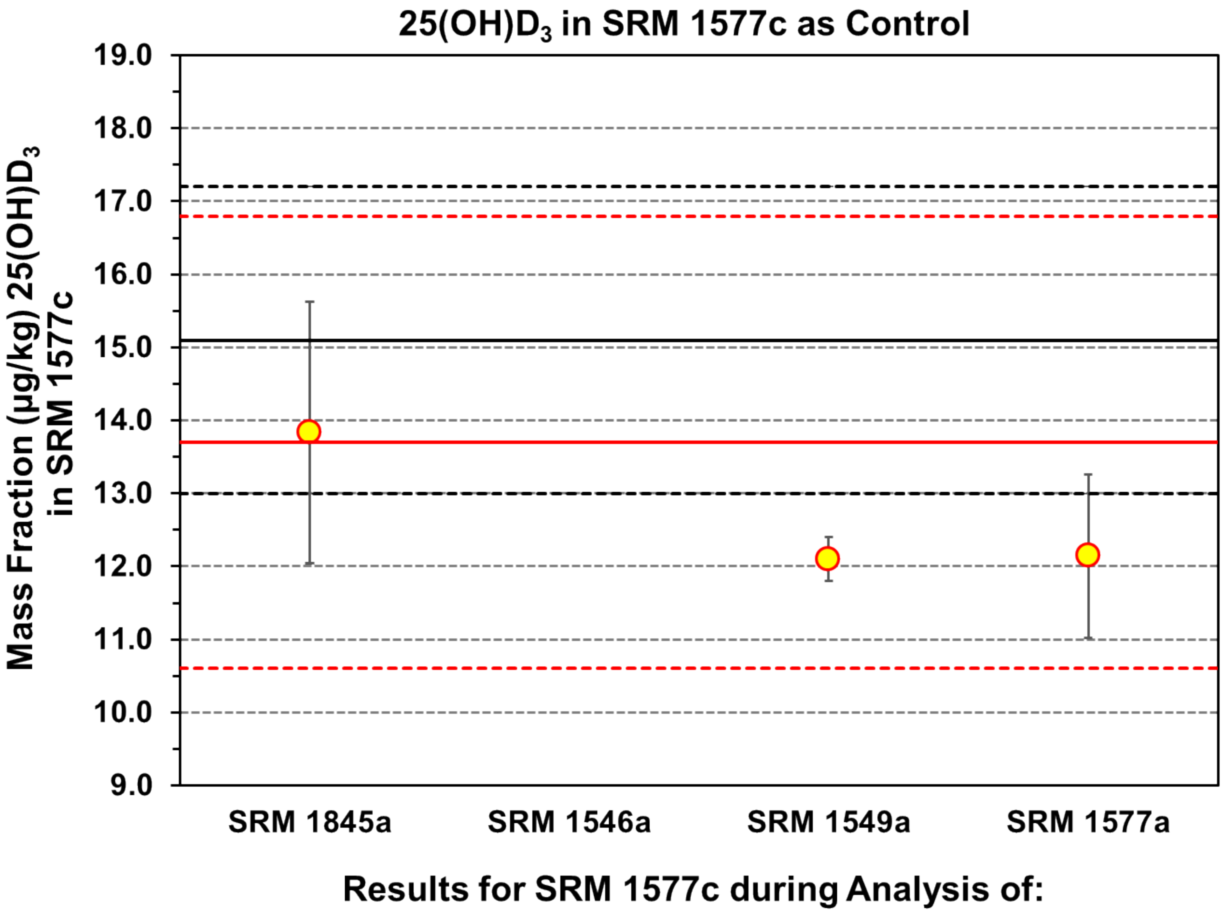

Figure S7. Results for the analysis of SRM 3280 Multivitamin/Multielement Tablets as control material during the analysis of SRM 3235 Soy Milk for the determination of vitamin D_2. Certified value for vitamin D_2 is solid black line and the uncertainty of the certified value is represented by the dashed red lines. Yellow circle represents the mean of the 4 measurements of SRM 3280 when analyzed concurrently with SRM 3235, and the error bars represent ±SD of the measurements.

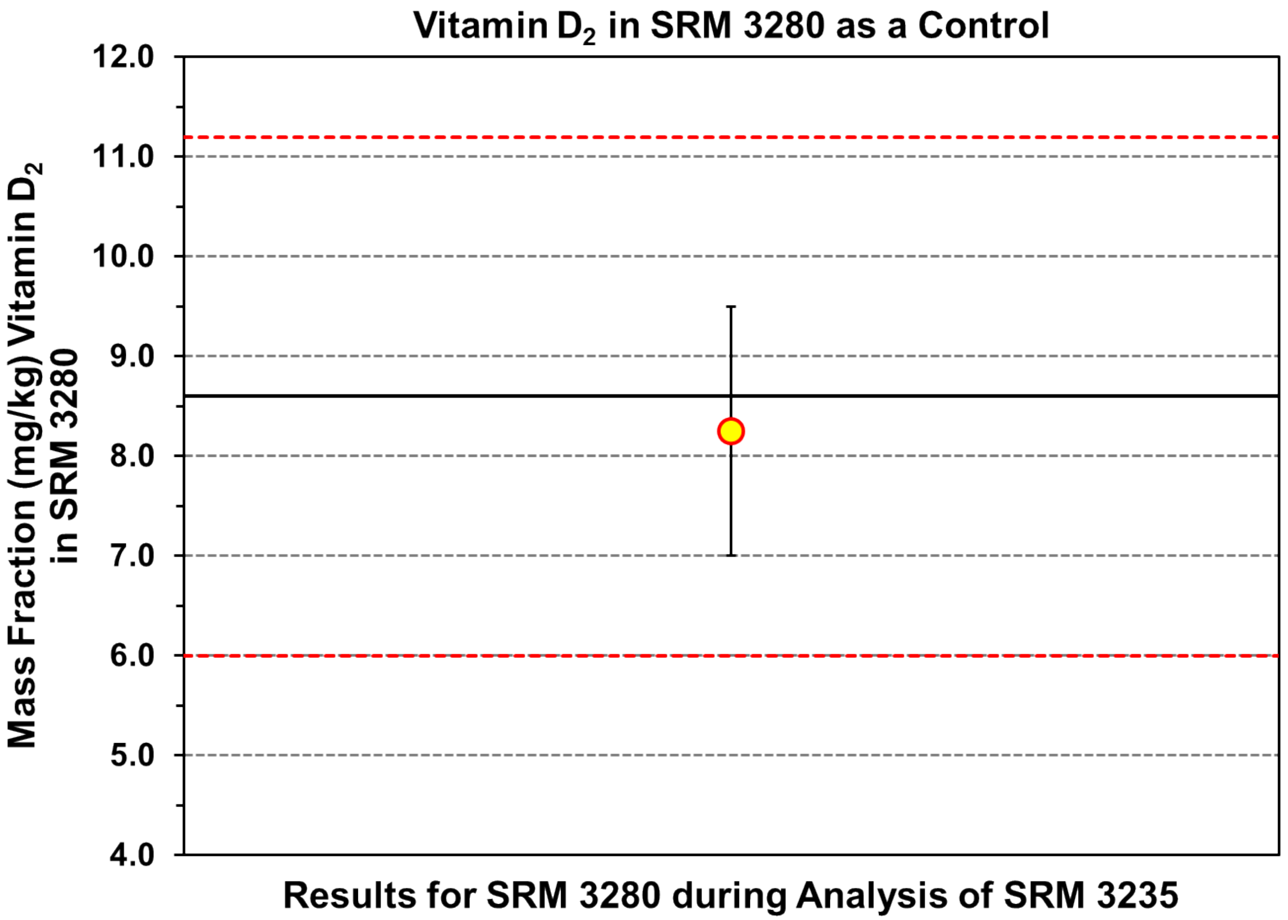

Supplement: Supplementary file 1 [file jf6c01298_si_001.pdf]
